# Analysis of species adulteration in beef sausage using real-time polymerase chain reaction in Makassar, Indonesia

**DOI:** 10.14202/vetworld.2024.2355-2364

**Published:** 2024-10-27

**Authors:** Mirna Mualim, Hadri Latif, Herwin Pisestyani, Puji Rahayu

**Affiliations:** 1 Graduate Program, Veterinary Biomedicine Study Program, Graduate School of IPB University, Jl. Agatis IPB Dramaga Campus, Bogor, Indonesia; 2 Faculty of Vocation, Study Program of Veterinary Paramedics, Hasanuddin University, Makassar, Indonesia; 3 Division of Veterinary Public Health and Epidemiology, School of Veterinary Medicine and Biomedicine, Jl. Agatis, Bogor, Indonesia; 4 Animal Products Quality Testing and Certification Center, Directorate General of Animal Husbandry and Animal Health, Ministry of Agriculture of the Republic of Indonesia, Tanah Sereal District, Bogor, Indonesia

**Keywords:** adulteration, beef sausage, deoxyribonucleic acid, polymerase chain reaction, species

## Abstract

**Background and Aim:**

Adulteration, or the inclusion of meats not declared on the label of processed meat products, constitutes a fraudulent practice that poses a threat to public health. Sausages, which are processed meats derived from a blend of minced meats that obscure the original muscle’s morphological features, are particularly prone to adulteration, making the visual detection of fraud more challenging. The research aimed to detect and measure the proportion of pork, chicken, buffalo, and beef DNA in commercially available processed meat packaged, labeled, and sold as “beef sausages” in Makassar, Indonesia.

**Materials and Methods:**

A total of 30 beef sausage samples were collected from traditional and modern markets as well as tourist attractions in Makassar. DNA was isolated and the species were identified using quantitative polymerase chain reaction.

**Results:**

The findings revealed that all sausage samples contained not only beef DNA, as indicated on their labels but also undeclared DNA from chicken and buffalo. Notably, pork DNA was not detected in the samples. The frequencies of chicken and buffalo meat were 9.2% and 10%, respectively, whereas it was 0.85% for beef in the beef sausage samples.

**Conclusion:**

The discovery of chicken and buffalo species in beef sausage samples indicates adulteration, potentially posing severe quality risks.

## Introduction

Meat and processed meat products are significant sources of high-quality proteins with essential amino acids and micronutrients vital to the human body [1]. Among the various types of meat, beef has a higher market price, making it a prime target for food fraud [2]. Given the cost differential, several animal species, such as pork, chicken, and buffaloes, which are significantly less expensive than beef, are potential adulterants in beef products [3, 4]. The most common forms of food fraud in animal-based products include counterfeiting, substituting, and mislabeling [5]. Food fraud is a pervasive global issue, data from the European Union Rapid Alert System for Food and Feed reveal that over a 20-year span (2000–2020), Indonesia ranked second in The Association of South-east Asian Nations for the highest number of export rejections due to food fraud and adulteration cases [6, 7]. In Indonesia, the government mandates that foods of animal origin must adhere to the ASUH criteria (Aman, Sehat, Utuh, dan Halal-Safe, Healthy, wholesome, and Halal) is a concept approved by the Indonesian Ministry of Agriculture to ensure the quality and safety of food products by adhering to specific standards. “Aman” means safe for consumption, “Sehat” refers to nutritional value, “Utuh” emphasizes that the product maintains its integrity without unwanted alterations, and “Halal” ensures compliance with Islamic dietary laws as regulated by the Indonesian Council of Ulama (Majelis Ulama Indonesia, MUI) before being distributed and consumed. This requirement is outlined in Government Regulation (PP) No. 22 of 1983 on veterinary public health, which was later superseded by Government Regulation (PP) No. 95 of 2012, incorporating animal welfare considerations [3]. This situation demands urgent attention from government bodies, regulatory agencies, the food industry, and society at large due to its implications for public health (e.g., allergies), religious practices, economic losses, diminished trust, and infringement of consumer rights [8].

Makassar, the largest city in Eastern Indonesia exhibits a notably high beef consumption rate, with an average of 15,000 tons/day, the highest in South Sulawesi Province. As the principal beef distribution hub in South Sulawesi, Makassar plays a critical role in the regional beef supply chain [9].

Approximately 85% of food fraud incidents involving processed meat, particularly beef products, involve adulteration [10]. This type of fraud typically involves the intentional mixing of meat from different species during the processing stage [11]. Sausages, which are made from ground meat, are especially prone to adulteration because the grinding process obscures the meat’s original morphological characteristics, making visual detection of fraud more challenging [8, 12]. Adulteration can have significant implications for public health, such as causing allergic reactions or food poisoning. It may also violate religious dietary restrictions, such as the prohibition of pork consumption for Muslims, and lead to substantial economic losses [3].

Species identification plays a crucial role in verifying the origin of food ingredients, which is essential for ensuring authenticity, integrity, and compliance with halal standards for food products [13, 14]. The extensive processing of meat products, including high-heat treatments, can lead to protein degradation, complicating species identification [8]. Deoxyribonucleic acid (DNA), as the carrier of genetic information from one generation to the next, maintains a stable structure and exhibits considerable resistance to temperature changes, making it detectable even in processed meat [15].

Real-time quantitative polymerase chain reaction (qPCR) is a DNA-based species identification technique that is notable for its high sensitivity, specificity, and efficiency [16, 17]. Recent studies have highlighted the effectiveness of qPCR in species identification in beef products. For instance, Cahyaningsari *et al*. [18] demonstrated qPCR’s capability to detect DNA using very low percentage-based standards, providing prompt results. Yörük [19] found that qPCR achieved a 100% success rate in species identification, significantly outperforming other methods such as enzyme-linked immunosorbent assay, which only achieved a 63.7% success rate. Given its ability to identify species in processed meat even in minute quantities, qPCR is increasingly considered a viable option for detecting food adulteration [20].

The adulteration of species in Makassar’s beef sausages remains unexplored. This study aimed to detect and measure the proportion of pork, chicken, buffalo, and beef DNA in commercially available processed meat, packaged, labeled, and sold as ‘beef sausages’ in Makassar, Indonesia.

## Materials and Methods

### Ethical approval

This study did not require ethical approval. Nonetheless, sample collection adhered to the established standards for sample collection procedures specified in SNI 3932: 2008 [21] and ISO 23854: 2021 [22]. Sausage samples were acquired from market vendors.

### Study period and location

The study was conducted from September 2023 to December 2023. DNA isolation [23] and species identification using qPCR method [4] were performed at the quality control laboratory and Certification of Animal Products, Ministry of Agriculture, Republic of Indonesia.

### Sample collection

Based on direct surveys conducted in traditional, modern, and tourist markets in Makassar City, 30 sausage vendors were identified. Therefore, we used a census method to represent all sausage vendors as sample units. The types of samples collected in this study were sausages labeled as beef products or reported to be made from 100% beef. Several criteria were used to categorize the sample that served as the object of research in supporting the research data. The sample in this study was the population that fulfilled the following criteria: (a) Inclusion criteria: Sausage samples made with beef without a combination of other meats; (b) exclusion criteria: Modern markets that were official markets registered with Perumda Pasar Makassar Raya and were located in the central area of Makassar city or directly adjacent to it; (c) traditional markets that operated at least once a week and were located in the downtown area of Makassar or directly adjacent to it; and (d) tourist attractions that were located in the Makassar city center area or directly adjacent to it. The distribution of sausage vendors and the number of samples per vendor is presented in Table-1. These 30 samples were transported to the laboratory at 4°C and stored at –20°C until further analyses [21, 22].

**Table-1:** Distribution of beef sausage vendors and number of samples around Makassar.

Group	Location name	Number of vendors	Number of samples
TM	TM 1	2	14
	TM 2	2	
	TM 3	1	
	TM 4	2	
	TM 5	1	
	TM 6	2	
	TM 7	2	
	TM 8	2	
MM	MM 1	2	10
	MM 2	3	
	MM 3	2	
	MM 4	3	
TA	TA 1	2	6
	TA 2	2	
	TA 3	2
Total samples			30

TM=Traditional markets, MM=Modern markets, TA=Tourist area

### DNA isolation

The sausage samples were initially homogenized in a blender. Subsequently, 25 mg of each sample was carefully transferred into a microtube. DNA was isolated from these sausage samples in strict adherence to the standardized protocols outlined in the Food Manual Book provided with the Quick-DNA™ Miniprep Kit (Zymo Research, Irvine, CA, USA). Following extraction, DNA isolates were stored at −20°C [23].

The concentration and purity of DNA isolates were checked using NanoDrop™ 2000c Spectrophotometers (Thermo Fisher Scientific Inc., Waltham, MA, USA). For this purpose, 2 μL of the DNA isolate was applied to a pre-calibrated NanoDrop™ 2000c spectrophotometer.

### Species identification

To determine the presence of DNA from pork, chicken, buffalo, and beef in the resulting sausage samples, specific primers and probes were used as described in Table-2 [4, 24]. The probes used in this study were selected based on their previously reported sensitivity and specificity for detecting species-specific genetic markers. According to Tanabe *et al*. [4] and Drummond *et al*. [24], the sensitivity and specificity of these probes were 100% for both when tested under controlled laboratory conditions. These metrics indicate the ability of the probes to accurately identify the presence of target DNA sequences without significant cross-reactivity. The amplification process commenced with the combination of 20 μL of reaction master mix (which included 12.5 μL of Fast Universal Master Mix Probe, 1 μL of both forward and reverse primers and 5.5 μL of nuclease-free water) with 5 μL of the DNA sample. The resulting mixture was transferred into a microtube, resulting in a total reaction volume of 25 μL. DNA amplification was executed using a Rotor-Gene Q thermal cycler (Qiagen, Hilden, Germany). To ensure accuracy, each real-time PCR assay included a positive control for each species being tested (i.e., pork DNA, chicken DNA, buffalo DNA, and beef DNA) as well as a negative control (nuclease-free water). The amplification protocol was performed over 45 cycles lasting 1 h, with specific temperatures and durations for each step: initial denaturation at 95°C for 5 min, denaturation at 95°C for 15 s, annealing at 60°C for 23 s, and elongation/extension at 72°C for 10 s. The real-time amplification process was monitored and displayed using the Q-Rex software v2.0 (https://www.qiagen.com/cn/resources/resourcedetail?id=b33be7c1-e9fb-488a-9e52-e8de673556e3$lang=en) on the Rotor-Gene Q thermal cycler (Qiagen, Hilden, Germany) [4, 25].

**Table-2:** Primers and probes used in this study.

Species	Primers and Probes	Nucleotide sequence (5’–3’direction)	References
Pig	F	CTTGCAAATCCTAACAGGCCTG	[4]
	R	CGTTTGCATGTAGATAGCGAATAAC	
	Probe	(FAM)-ACAGCTTTCTCATCAGTTAC-(NFQ) (MGB)	
Chicken	F	CTGGGCTTAACTCTCATACTCACC	[4]
	R	GGTTACTAGTGGGTTTGCTGGG	
	Probe	(FAM)-CATTCCTAACACTAGCCCTA-(NFQ) (MGB)	
Buffalo	F	TCAGCCCAAAGAAAAATAAACCA	[24]
	R	GTCACCCCAACCGAAACTGT	
	Probe	(FAM) TAAGGARTAACAACAMTCT-MGB	
Cattle	F	CCCGATTCTTCGCTTTCCAT	[4]
	R	CTACGTCTGAGGAAATTCCTGTTG	
	Probe	(FAM)-AGTGGCAGACTTACTG-(NFQ) (MGB)	

F=Forward Primer, R=Reverse primer

### Quantification of meat composition using a standard curve

Positive results from species identification tests were followed by quantification tests of each species’ meat composition in the sausages using qPCR, following Tanabe *et al*. [4] with modifications in percentage of concentration for each species. To ensure the reliability of the probes, quality control testing was performed on unadulterated chicken, buffalo, and beef meat samples using the standard curve method. DNA was extracted from known quantities of pure meat from each species, and samples were mixed to create percentage-based standards for each species. Chicken DNA was designed using percentage-based standards of 20%, 10%, 5%, 2.5%, and 1.25%. Buffalo DNA was designed using percentage-based standards of 40%, 20%, 10%, 5%, and 2.5%. Beef DNA was designed at concentrations of 2.5%, 1.25%, 0.625%, 0.3125%, and 0.15625% [4, 24].

The meat concentration of each positive species was estimated based on the DNA concentration from the cycle threshold (Ct) values obtained from the species identification amplification curve. Quantification of meat composition from positive species referred to a standard curve created from the Ct values of meat testing at five serial percentage-based standards. The standard curve was generated by plotting the Ct values from quantitative PCR (qPCR) against the logarithm of the DNA concentration percentage of each species. This percentage-based approach allowed the precise determination of probe linearity, efficiency, and correlation. The qPCR results produced a standard curve with high linearity (R² > 0.99). Efficiency calculations were performed using the equation [26]:

E = 10 ^(*-1/slope*)^ and percentage efficiency = (E-1) × 100

The standard curve served as a reference for calculating the percentage of meat content from each species in beef-labeled sausages.

### Statistical analysis

The data were analyzed descriptively and presented in the tables and figures.

## Results

### Quality control of DNA

The quality control results for the DNA from the beef sausage samples indicated that the DNA was successfully isolated. The concentrations and purity of DNA from the sausage samples are presented in Table-3.

**Table-3:** DNA concentrations and DNA purity of beef sausage samples determined using a spectrophotometer nanodrop.

n = 30	DNA concentration (ng/μL)	DNA purity	

		A260/280	A260/230
Mean value ± SD	42.84 ± 18.09	1.90 ± 0.05	1.83 ± 0.18
Minimum values	18.40	1.81	1.63
Maximum values	93.20	2.03	2.26

DNA=Deoxyribonucleic acid, SD=Standard deviation

Table-3 shows the mean DNA concentration of 42.84 ± 18.09 ng/μL, with a range from 18.40 ng/μL to 93.20 ng/μL, indicating that the DNA concentration is adequate for PCR processing as percentage-based standards above 10 ng/μL are suitable for such analysis [27]. The purity of beef sausage DNA samples, as measured by the A260/280 ratio, averaged 1.90 ± 0.05, with the lowest value at 1.81 and the highest at 2.03. According to Matlock [27], DNA purity values between 1.8 and 2.0 indicate good quality. The average purity of beef sausage DNA was generally satisfactory. Purity values above 2.0 can occur when reagents such as phenol, alcohol, and chloroform are inadvertently coextracted with DNA, affecting the absorbance results [27–29].

The A260/230 ratio is a highly sensitive indicator of contaminants. Many contaminants are absorbed at the 230 nm wavelength, including guanidine thiocyanate (GTC), guanidine hydrochloride, ethylenediaminetetraacetic acid, phenols, proteins, and polysaccharides [30]. The average A260/230 purity value for beef sausage samples in Makassar City was 1.83 ± 0.18, with the lowest at 1.63 and the highest at 2.26. The standard purity range for A260/230 lies between 2.0 and 2.2 [26, 29]. Most beef sausage samples showed low DNA purity, potentially due to GTC contamination during extraction, which maximally absorbed at 230-nm wavelengths, thereby reducing the DNA purity ratio A260/230 [29, 30]. Furthermore, as sausages are processed products, they are more likely to contain contaminants than fresh meat samples. However, according to Widayat *et al*. [31], substandard A260/230 purity values do not significantly affect qPCR amplification, allowing samples to be used for further qPCR analyses.

### Identification of species in beef sausage

**Figure-1:**
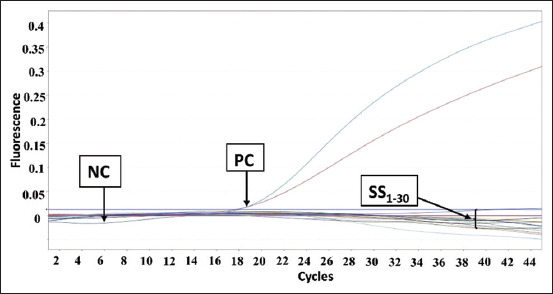
Results of polymerase chain reaction amplification of beef sausage samples sold in Makassar with pork primers. PC=Positive control pork deoxyribonucleic acid (DNA), NC=Negative control (no template DNA), samples were coded with numbers SS1-SS30.

**Figure-2:**
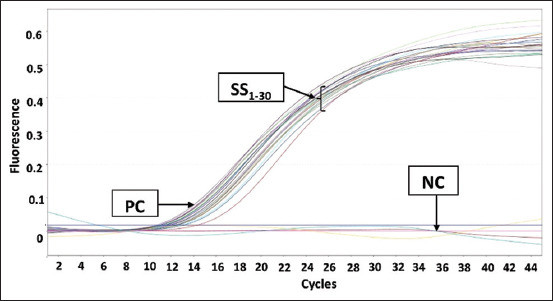
Results of polymerase chain reaction amplification of beef sausage samples sold in Makassar with chicken primers. PC=Positive control chicken deoxyribonucleic acid (DNA), NC=Negative control (no template DNA), samples were coded with numbers SS1-SS30.

**Figure-3:**
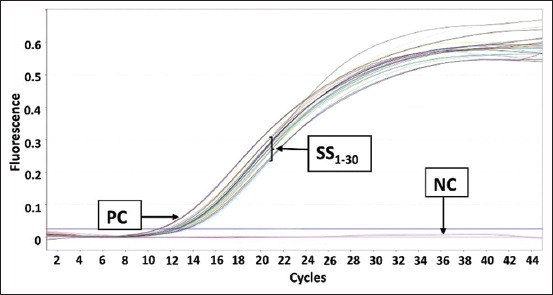
Results of polymerase chain reaction amplification of beef sausage samples sold in Makassar with buffalo primers. PC=Positive control buffalo deoxyribonucleic acid (DNA), NC=Negative control (no template DNA), samples were coded with numbers SS1-SS30.

**Figure-4:**
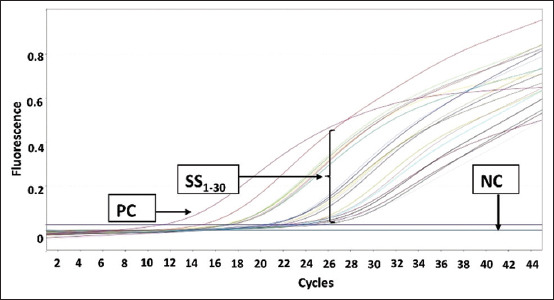
Results of polymerase chain reaction amplification of beef sausage samples sold in Makassar with beef primers. PC=Positive control beef deoxyribonucleic acid (DNA), NC=Negative control (no template DNA), samples were coded with numbers SS1-SS30.

The PCR amplification results for pork DNA from beef sausage samples in Makassar are shown in Figure-1. The results showed that there was no amplification in any of the samples. Figure-2 shows the amplification results for beef sausage samples from Makassar City using chicken DNA primers, revealing that all of the beef sausage samples contained chicken DNA. Figure-3 presents the amplification results for beef sausage samples using buffalo DNA primers; all samples contained buffalo DNA. The PCR amplification results for bovine DNA from beef sausage samples are shown in Figure-4, indicating that all beef sausage samples contained beef DNA.

Specifically, in the amplification of pork DNA (Figure-1), only the positive control (pork DNA) exhibited an amplification process, mirroring the lack of amplification observed in all sausage samples, which was identical to the negative control (nuclease-free water). The amplification curves for chicken DNA (Figure-2), buffalo DNA (Figure-3), and beef DNA (Figure-4) on the other hand showed a clear rise, going over the threshold and forming a sigmoid curve, indicating that the amplification was successful. The amplification processes for chicken, buffalo, and beef DNA in all samples yielded Ct values below 35, as detailed in Table-3, confirming the presence of these DNA types in all sausage samples.

The amplification of chicken DNA (Figure-2) and buffalo DNA (Figure-3) reached the threshold significantly quicker than that of beef DNA (Figure-4). This observation suggests that the concentration of target DNA in a sample is directly proportional to the speed at which it reaches the threshold, with higher percentage-based standards leading to faster threshold attainment. Beef sausage samples contained higher levels of chicken and buffalo DNA than beef DNA [21]. The mean Ct values for sausage samples with chicken and buffalo DNA were 12.50 and 12.30, respectively. This result differed from that of beef DNA, which had a mean Ct value of 21.70. According to Bonacorsi *et al*. [32], lower Ct values in qPCR assays indicate a higher DNA concentration within the sample, whereas higher Ct values indicate a lower DNA concentration. This suggests that the percentage-based standards of chicken and buffalo DNA in beef sausage samples are substantially higher than that of beef DNA. This finding is particularly notable given that beef DNA is the only species declared on the packaging of sausage products, indicating a discrepancy between the product’s labeling and its actual DNA content.

### Quantification of meat composition using a standard curve

Three animal species (chicken, buffalo, and beef) those tested positive in the samples labeled “beef sausages” were further tested to estimate the mean concentration of each species in the samples. The results of qPCR testing on chicken, buffalo, and beef meat according to five different percentage-based standards are presented in Table-4.

**Table-4:** Cycle thresholds for pork, chicken, buffalo, and cattle detected using qPCR.

Sample code	Pork	Ct value	Chicken	Ct value	Buffalo	Ct value	Beef	Ct value
SS 1	-	No Ct	+	12.65	+	12.64	+	23.80
SS 2	-	No Ct	+	13.59	+	13.53	+	25.10
SS 3	-	No Ct	+	12.23	+	12.10	+	20.71
SS 4	-	No Ct	+	12.70	+	11.68	+	16.52
SS 5	-	No Ct	+	11.97	+	12.60	+	17.39
SS 6	-	No Ct	+	13.03	+	13.20	+	21.83
SS 7	-	No Ct	+	11.33	+	12.50	+	24.24
SS 8	-	No Ct	+	11.28	+	10.95	+	15.99
SS 9	-	No Ct	+	13.86	+	13.37	+	16.34
SS 10	-	No Ct	+	11.99	+	12.09	+	16.97
SS 11	-	No Ct	+	11.69	+	10.98	+	24.05
SS 12	-	No Ct	+	13.20	+	12.99	+	17.33
SS 13	-	No Ct	+	13.09	+	12.41	+	16.85
SS 14	-	No Ct	+	12.74	+	11.54	+	17.23
SS 15	-	No Ct	+	11.74	+	12.41	+	21.78
SS 16	-	No Ct	+	12.85	+	12.88	+	21.16
SS 17	-	No Ct	+	12.76	+	12.62	+	21.23
SS 18	-	No Ct	+	11.41	+	11.76	+	22.37
SS 19	-	No Ct	+	13.28	+	12.42	+	20.08
SS 20	-	No Ct	+	14.56	+	12.66	+	25.45
SS 21	-	No Ct	+	12.38	+	12.00	+	30.43
SS 22	-	No Ct	+	12.59	+	11.80	+	23.36
SS 23	-	No Ct	+	11.48	+	12.19	+	22.36
SS 24	-	No Ct	+	12.77	+	12.58	+	20.55
SS 25	-	No Ct	+	12.11	+	11.96	+	25.29
SS 26	-	No Ct	+	12.27	+	12.50	+	25.43
SS 27	-	No Ct	+	12.66	+	12.02	+	30.40
SS 28	-	No Ct	+	12.07	+	11.70	+	23.32
SS 29	-	No Ct	+	12.16	+	12.18	+	22.30
SS 30	-	No Ct	+	11.69	+	12.50	+	20.51
Mean values				12.50		12.30		21.70
Minimum values				11.28		10.95		15.99
Maximum values				14.56		13.53		30.43

qPCR=quantitative polymerase chain reaction, Ct=Cycle threshold

Based on the results of the qPCR analysis, standard curves for chicken DNA designed using percentage-based standards of 20%, 10%, 5%, 2.5%, and 1.25% yielded Ct values of 11.09, 12.16, 13.21, 14.40, and 15.41, respectively. Standard curves for buffalo DNA designed using percentage-based standards of 40%, 20%, 10%, 5%, and 2.5% yielded Ct values of 10.21, 11.15, 12.10, 13.07, and 14.07, respectively. Standard curves for beef DNA designed using percentage-based standards of 2.5%, 1.25%, 0.625%, 0.3125%, and 0.15625% yielded Ct values of 16.20, 17.39, 18.40, 19.30, and 20.50, respectively. The results show that higher percentage-based standards in all three standard curves result in lower Ct values. Theoretically, lower Ct values indicate a sample contains a high concentration of target DNA, with Ct values <35 [19, 32, 33].

**Figure-5:**
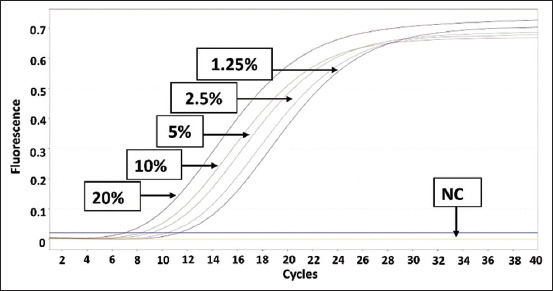
Polymerase chain reaction amplification of the chicken standard curve. NC=Negative control.

**Figure-6:**
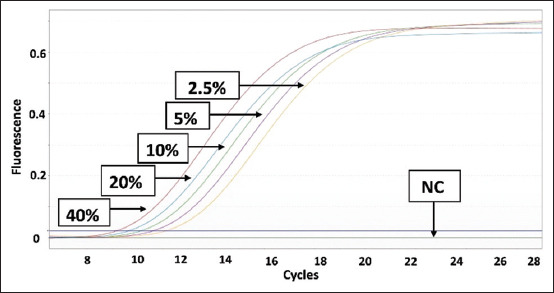
Polymerase chain reaction amplification of the buffalo standard curve. NC=Negative control.

**Figure-7:**
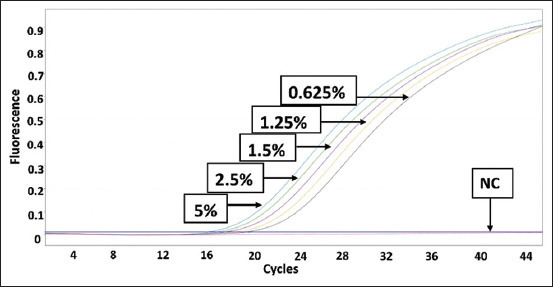
Polymerase chain reaction amplification for a beef standard curve. NC=Negative control.

**Figure-8:**
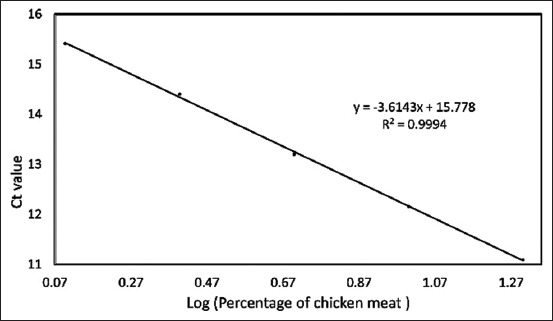
Linear regression equation graph for the chicken standard curve. Ct=Cycle threshold, Log=Logarithm, y=Linear regression equation, R^2^=Efficiency.

**Figure-9:**
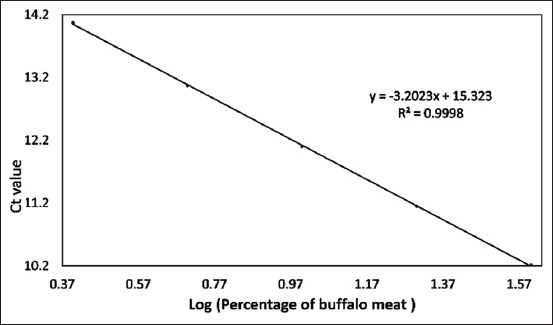
Linear regression equation graph for the buffalo standard curve. Ct=Cycle threshold, Log=Logarithm, y=Linear regression equation, R^2^=Efficiency.

**Figure-10:**
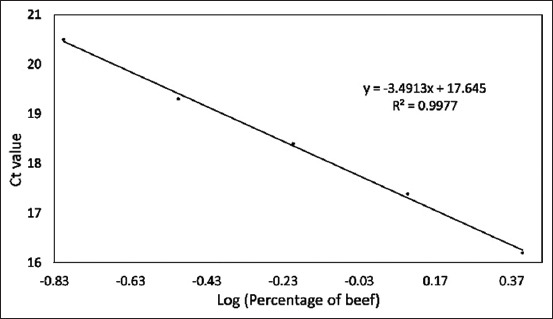
Linear regression equation graph for the beef standard curve. Ct=Cycle threshold, Log=Logarithm, y=Linear regression equation, R^2^=Efficiency.

Data from Table-4 can be graphed as a linear regression equation by plotting the log of the concentration of each species on the X-axis and the Ct values from the qPCR analysis on the Y-axis. This section is intended to evaluate the linearity and accuracy of the standard curves before using them to measure sample percentage-based standards. The amplification curves for the three standard curves are shown in Figures-5–7. The linear regression graphs for the three standard curves are shown in Figures-8–10. The detection system demonstrated that the primers used in this study functioned well. This trend was evident from the exponential increase in the amplification curves for chicken (Figure-5), buffalo (Figure-6), and beef (Figure-7) species according to the dilution percentage. The entire amplification process occurred before cycle 35. Subsequently, the three standard curves were evaluated for linearity and sensitivity by creating graphs that plotted the Ct values from the standard curves on the X-axis against the log concentration values of the dilutions from the standard curves on the Y-axis. The linear regression equations for chicken (Figure-8), buffalo (Figure-9), and beef DNA (Figure-10) standard curves were obtained as y = −3.6143x + 15.778, y = −3.2023x + 15.323, and y = −3.4913x + 17.645, respectively. The results showed that the standard curves for chicken, buffalo, and beef DNA had slopes of 3.6143, 3.2023, and –3.4913, respectively. The efficiency values were calculated as follows from the slopes of these three standard curves: E = (10^(-1/*slope*^) × 100). The results indicated that the correlation coefficients (R^2^) and efficiency values for the standard DNA curves were as follows: Chicken DNA 0.9994, efficiency of 90.6%; buffalo DNA 0.9998, efficiency of 105%; and beef DNA 0.9977, efficiency of 102%. The slope values of the three standard curves fell within the expected slope range of 3.1–3.6; hence, the efficiency values obtained were also good, ranging from 90% to 105% [26]. According to Pestana *et al*. [33], a good standard curve should have efficiency values greater than 80% and a slope between 3.1 and 3.6. Figures-8–10 show that the three standard curves have linear tangents and that almost all points lie on a single linear line. The R^2^ values obtained from the three curves were in the range of 0.9 ≤ R^2^ ≥ 1, or the R^2^ values approached 1 [26]. These results indicated a strong correlation between the concentration from the standard curve and the Ct values, demonstrating high accuracy [26, 34]. Therefore, we conclude that the three designed standard curves exhibit high linearity and accuracy, making them suitable for calculating sample percentage-based standards.

### Quantification of meat content in beef sausage samples

The previous qPCR analysis to detect the presence of animal species in beef sausages circulating in Makassar showed positive results for three species: Chicken, buffalo, and beef. Subsequent analysis was conducted using standard curves that had been sensitivity-tested to estimate the meat composition in the sausage samples. The meat composition of the samples was determined by comparing the standard curves and Ct values of the beef sausage samples for the three positively identified species. The percentages of chicken, buffalo, and beef meat in the sausage samples are presented in Table-5.

**Table-5:** Percentage composition of chicken, buffalo, and beef in beef sausage samples.

Species	Species composition (%)		

	Mean (n = 30)	Min. values	Max. values
Chicken	9.2	1.92	19.09
Buffalo	10.01	3.9	22.1
Beef	0.85	0.15	2.47

Table-5 displays the percentage composition of the three species obtained by comparing Ct values of the beef sausage samples (Table-4) with the Ct values of the standard curves for each species (Table-6). The results indicated that buffalo meat had the highest content, at 10%, in beef-labeled sausage samples. This content was not significantly different from the chicken meat content (9.2%). The beef sausage samples contained only 0.85% beef. Beef was expected to be the primary and sole animal ingredient in the sausages; however, the study revealed that all beef sausage samples contained <1% beef.

**Table-6:** Concentrations of chicken, buffalo, cattle, and Ct values determined using qPCR.

Species	Meat concentration (%)	Ct value
Chicken	20	11.09
	10	12.16
	5	13.20
	2,5	14.40
	1,25	15.41
Buffalo	40	10.21
	20	11.15
	10	12.10
	5	13.07
	2,5	14.07
Cattle	2,5	16.20
	1,25	17.39
	0,625	18.40
	0,3125	19.30
	0,15625	20.50

qPCR=quantitative polymerase chain reaction, Ct=Cycle threshold

## Discussion

Species identification plays a crucial role in mitigating adulteration in processed meat products. The adulteration of foods of animal origin can significantly impact public health (e.g., allergies), economic integrity, and religious observances [35–37].

The ASUH concept framework in Indonesia dictates that animal-based foods must be free from biological, chemical, and physical hazards (safe), nutritionally balanced (healthy), not adulterated with parts of other animals, true to label claims (whole), and processed according to Islamic law (halal). Therefore, the identification of chicken and buffalo DNA in beef sausage samples indicates a breach of the “whole” principle of the ASUH criteria, as the products contained unlisted animal species [33].

Chicken meat in beef sausage samples from this study was found to be 9.2%. The substitution of chicken meat in beef products is a widespread practice in both developing and developed nations, including Indonesia [36]. Research by Siswara *et al*. [3] found that 35/36 beef meatballs in Boyolali contained chicken DNA. Similarly, Wibowo *et al*. [38] found that 27/33 (81.8%) processed beef products in Bogor and Surakarta were adulterated with chicken DNA. These findings are likely driven by the lower cost of chicken meat, as reported by the National Food Agency of Indonesia [39], compared to beef, as well as its availability. According to Li *et al*. [40], such adulteration often stems from Economically Motivated Adulteration (EMA), where producers, driven by profit, use or mix cheaper meat without proper labeling.

Buffalo meat was also found to be 10% in the tested beef sausage samples. In contrast, with the relatively high content of buffalo DNA, beef, which is the main ingredient listed on the packaging label, was only about <1%. Keyvan *et al*. [13] highlighted the economic reasons behind the incorporation of buffalo meat into beef products, noting the significant price difference and the physical resemblance between buffalo meat and beef, which facilitates adulteration [41]. According to the National Food Agency of Indonesia [39], beef prices are twice as high as those of buffalo meat, underscoring the economic incentive behind the observed adulteration with buffalo DNA in the study.

No pork DNA was not found in the beef sausage samples. The rigorous regulation and oversight of pork distribution in Indonesia, where the majority of the population follows Islam, likely contributed to this outcome. In addition, data from Statistics Indonesia [42] indicate that pork production in Makassar is lower than in other regions, further reducing the likelihood of pork being present in beef products. When pork is found in processed beef products, the quantities are typically very small (<0.1%), suggesting accidental contamination during manufacturing rather than intentional adulteration, as minimal amounts of pork would not yield significant economic benefits to producers [43, 44].

Mislabeling of beef products is a form of food fraud, where products are inaccurately labeled [45]. Indonesian legislation, specifically Article 97 of Law No. 18 of 2012 concerning food, mandates accurate labeling of food products, including the product name and a list of ingredients [3]. Furthermore, Law No. 18 of 2012 by the Food and Drug Supervisory Agency on processed food labels requires the inclusion of the percentage of raw material content on the label [38]. The findings of this study highlight an issue of mislabeling: 30 sausage samples labeled as beef failed to disclose the inclusion of chicken and buffalo DNA in their ingredient list. This issue is not unique to Indonesia; similar mislabeling cases have been documented globally. Naaum *et al*. [46] found that 20% of sausages in Canada were mislabeled by omitting the meat content of other species. In Malaysia, Chuah *et al*. [47] reported 112 (78.3%) instances of mislabeled processed beef product samples, and Song *et al*. [1] identified 64 beef sausage samples with incorrect labels in Sichuan Province, China.

The significance of accurate labeling on processed product packaging cannot be overstated, particularly for consumers with food allergies, as accurate labeling is a key factor in their product selection process. Accurate labels are essential to avoid allergens that can cause serious health problems. For instance, individuals allergic to chicken may experience symptoms ranging from mild-to-severe itching and hand edema [48]. In some cases, chicken allergies can also induce urticaria and asthma [37, 49]. Similarly, buffalo meat allergies can manifest as oral itching due to urticaria [48].

The real-time polymerase chain reaction (PCR) method can identify species in small amounts of meat food, making it an alternative to find food adulteration [20]. Many studies have been conducted to develop efficient adulteration detection methods. However, most current studies only detected adulteration and did not measure the amount of material used [34]. Measuring the DNA concentration of other species in meat products can be an important factor in developing an adulteration hypothesis [50]. This study used a standard curve, also known as a calibration curve, which is a curve made to estimate the range of DNA percentage-based standards in samples [32, 51]. The standard curve allows the production of specific, sensitive, and accurate real-time PCR data. This method is considered the appropriate method for measuring DNA samples in qPCR [26]. However, qPCR has the limitation that its screening costs are more expensive compared to other screening methods [52].

## Conclusion

Beef sausages circulating in Makassar contained not only beef DNA, as declared on the product label, but also undeclared species such as chicken and buffalo DNA. Notably, no pork DNA was detected in the tested samples. The discovery of chicken and buffalo species in beef sausage samples indicates the adulteration of beef sausages, potentially posing serious risks to public health and economic losses. This series of studies underscores the importance of authenticity and integrity in animal-based foods. Therefore, recommendations include: (1) Providing educational initiatives for business operators regarding adulteration and labeling practices in animal-based processed foods, (2) implementing effective monitoring and oversight by the local government of Makassar regarding the distribution of animal-based foods, and (3) raising consumer awareness about the importance of choosing registered products.

## Authors’ Contributions

MM, HL, and HP: Conceptualized the study and methodology. PR, HP, and HL: Performed the validation. HL and HP: Performed the formal analysis. MM and PR: Investigation. HL, MM, and HP: Interpreted the data and drafted and revised the manuscript. MM: Project administration. All authors have read and approved the final manuscript.
